# Application Value of Combined Detection of NLR, PNI, D-Dimer, CD3^+^ T Lymphocytes, and CEA in Colorectal Cancer Screening

**DOI:** 10.1155/2022/7913025

**Published:** 2022-03-20

**Authors:** Rui Ding, Zheng Chen, Ming He, Hong Cen, Zehui Liu, Yonghui Su

**Affiliations:** Department of Gastrointestinal Surgery, The Fifth Affiliated Hospital of Sun Yat-Sen University, Zhuhai, Guangdong 519000, China

## Abstract

**Objective:**

To investigate the application value of combined detection of neutrophil-lymphocyte ratio (NLR), prognostic nutrition index (PNI), D-dimer (D-D), CD3^+^ T lymphocytes (CD3^+^ T), and carcinoembryonic antigen (CEA) in colorectal cancer screening.

**Methods:**

The study cohort comprised 187 colorectal cancer patients and 100 mixed hemorrhoids patients as controls from January 2019 to August 2021 at the Fifth Affiliated Hospital of Sun Yat-sen University. Comparing the levels of NLR, PNI, D-D, CD3^+^ T, and CEA between the two groups of subjects, drawing receiver operating characteristic (ROC) curve evaluates the efficacy of single and combined detection for colorectal cancer screening.

**Results:**

Compared with the control group, the levels of NLR, D-D, and CEA in the colorectal cancer group were significantly increased, while the levels of PNI and CD3^+^ T were significantly decreased (*P* < 0.05). ROC curve analysis showed that the combined detection of NLR, PNI, D-D, CD3^+^ T, and CEA for colorectal cancer screening had an AUC^ROC^ of 0.943, a sensitivity of 84.49%, a specificity of 91.00%, and a Youden index of 0.75, and its screening efficacy was significantly superior to that of a single detection (*P* < 0.001).

**Conclusion:**

The combined detection of NLR, PNI, D-D, CD3^+^ T, and CEA has a high clinical application value for colorectal cancer and can provide a reference for early screening and auxiliary diagnosis of colorectal cancer.

## 1. Introduction

Colorectal cancer (CRC) is the most common gastrointestinal malignancy that seriously harms human health in the world, ranking third in morbidity and second in mortality [1]. The onset of colorectal cancer is relatively insidious, and there are no obvious specific symptoms. A considerable number of patients are often in the middle and advanced stages of the disease when they are diagnosed. However, the “gold standard” of diagnosis-colonoscopy pathological biopsy has limitations such as invasiveness, low acceptance by the population, and long examination time, and imaging examinations such as computed tomography (CT) and magnetic resonance imaging (MRI) also have the disadvantages of radioactivity and high cost [2–4]. Therefore, it is of great clinical significance to find simple, noninvasive, economical, and highly acceptable clinical screening indicators to improve the detection rate of colorectal cancer patients.

Carcinoembryonic antigen (CEA) is the most commonly used tumor marker for the clinical screening and diagnosis of colorectal cancer. It has the advantages of simple operation, noninvasiveness, and good reproducibility, but due to insufficient sensitivity and low organ specificity, its single detection has certain limitations. Recently, an increasing number of studies have shown that inflammation plays a very important role in the occurrence and development of tumors [5]. As common evaluation indicators of systemic inflammation, the neutrophil-lymphocyte ratio (NLR) and prognostic nutrition index (PNI) have been considered to have certain value in the diagnosis and prognosis of colorectal cancer [6–8]. D-dimer (D-D) is a specific degradation product produced when crosslinked fibrin is degraded by plasmin. It has been reported that patients with digestive system malignant tumors are more likely to develop thrombosis and form disseminated intravascular coagulation when D-D is elevated [9] . A high D-dimer level is closely related to poor prognosis of colorectal cancer, but there are few studies on its screening value for colorectal cancer [10]. Cellular immunity mediated by T lymphocyte subsets is the main method of the body's antitumor immunity in which the number of CD3+ T lymphocytes (CD3+ T) represents the total cellular immunity status of the body. Some studies have shown that an increase in the number of T lymphocyte subsets is associated with a good prognosis of colorectal cancer, and the change in the number of CD3^+^ T may be a clinical biomarker for auxiliary diagnosis [11]. A blood testing is an easily accepted habitual diagnosis and treatment item that naturally has the advantages of simplicity, noninvasiveness, economy, etc., and the above five indicators can all be obtained through clinical blood testing. Single indicator screening for colorectal cancer has the problem of low sensitivity or specificity. To improve the comprehensive ability of tumor screening and diagnosis, a combination of multiple indicators is usually adopted.

Therefore, this study is aimed at exploring the application value of the combined detection of NLR, PNI, D-D, CD3^+^ T, and CEA in colorectal cancer to provide a reference for the early screening and auxiliary diagnosis of colorectal cancer.

## 2. Materials and Methods

### 2.1. General Information

A retrospective analysis of 187 patients with colorectal cancer who were diagnosed and treated at the Fifth Affiliated Hospital of Sun Yat-sen University from January 2019 to August 2021 (study group) included 116 males and 71 females, with a median age of 63 years (range, 26-88). Inclusion criteria were as follows: (1) patients with complete clinicopathological data, (2) patients with primary colorectal cancer who underwent radical resection of colorectal cancer, and (3) patients with colorectal cancer confirmed by preoperative colonoscopy or postoperative gross histopathology. Exclusion criteria were as follows: (1) patients with or had other malignant tumors at the same time; (2) combined with severe cardiovascular and cerebrovascular diseases, severe liver and kidney dysfunction, severe blood, and rheumatic immune system diseases; (3) previously received neoadjuvant radiotherapy and chemotherapy and related anticancer drug therapy; (4) patients who have clear clinical evidence of infection within 2 weeks before admission or use anti-inflammatory, hormonal, or immunosuppressive drugs; and (5) patients who have received blood transfusion within 1 month. A total of 100 patients with nontumor mixed hemorrhoids (control group) were selected in the same period, including 57 males and 43 females, with a median age of 38 years (range, 23-69). Inclusion criteria were as follows: laboratory examinations such as blood routine, liver and kidney function and coagulation function tests, and imaging examinations such as chest X-ray fluoroscopy, color Doppler ultrasound, and electrocardiogram were complete and without obvious abnormalities. Exclusion criteria were as follows: (1) patients with or had suffered from malignant tumors or precancerous lesions at the same time; (2) combined with severe cardiovascular and cerebrovascular diseases, severe liver and kidney dysfunction, severe blood, and rheumatic immune system diseases; and (3) patients who had received blood transfusion within 1 month. This study was approved by the Medical Ethics Committee of our hospital.

### 2.2. Research Methods

Basic information and clinical data of subjects in the two groups were collected, including age, sex, past history, diagnosis, peripheral blood routine (neutrophil count, lymphocyte count), serum albumin, D-dimer, CEA, and CD3^+^ T. The NLR and PNI values were calculated as follows: NLR = neutrophil count (10^9^/L)/lymphocyte count (10^9^/L); PNI = total lymphocyte count (10^9^/L) × 5 + serum albumin (g/L) [12]. Postoperative pathological data of patients with colorectal cancer were collected, including tumor size, tumor location, histological type, differentiation degree, nerve invasion, vascular invasion, T stage, N stage (lymph node metastasis), and TNM stage (American Joint Committee on Cancer Staging Manual, 8th edition). Preoperative imaging results such as whole-abdominal enhanced CT and pelvic enhanced MRI were used to determine whether the primary tumor had distant metastasis.

The NLR, PNI, D-D, CD3^+^ T, and CEA levels were compared between the two groups, and receiver operating characteristic (ROC) curves were drawn to evaluate the screening efficacy of single and combined detection of these five indicators for colorectal cancer. According to the optimal cut-off values determined by the ROC curve, colorectal cancer patients were divided into high and low groups, and their relationship with clinicopathological parameters was analyzed. Logistic regression analysis was used to evaluate the relationship between the five indicators and colorectal cancer risk.

### 2.3. Statistical Analysis

The data were analyzed by SPSS 25.0 statistical software and MedCalc 20.0.9 software. Descriptive statistics were computed for all variables. Continuous data are presented as the mean ± SE or median (interquartile range: 25th and 75th percentiles), and categorical variables are presented as proportions (percentages). The Shapiro–Wilk test was used to test normally distributed continuous variables. Student's *t*-test or *U* test was used to analyze continuous variables depending on the normality of data distribution, and the *χ*^2^ or Fisher's exact test was used for categorical variables. The ROC curve was drawn by using MedCalc20.0.9 software to determine the area under the curve (AUC^ROC^), sensitivity, specificity, and Youden index under the optimal cut-off value. Logistic regression analysis in SPSS was used to evaluate the relationship between the five indicators and the risk of colorectal cancer. A two-sided *P* value <0.05 was considered to denote statistical significance for all tests.

## 3. Results

### 3.1. Comparison of NLR, PNI, D-D, CEA, T Lymphocyte Subsets, and Related Basic Data between the Two Groups

The colorectal cancer group was older (66 vs. 38 years old), and the levels of NLR (2.15 vs. 1.69), D-D (139 vs. 58 ng/ml), CEA (4.90 vs. 1.57 ng/ml), and CD8^+^T (732 vs. 532 *μ*L^−1^) were significantly higher than those in the control group, while the levels of CD3 + T (728 vs. 1332 *μ*L^−1^), CD4^+^T (612 vs. 868 *μ*L^−1^), CD4^+^/CD8^+^ ratio (1.34 vs. 1.53), and PNI (49.4 vs. 52.8) were significantly lower than those in the control group (*P* < 0.05). There was no significant difference in sex between the two groups (*P* > 0.05, Table 1).

### 3.2. Comparison of Single and Combined Detection in CRC Screening

**Figure 1:**
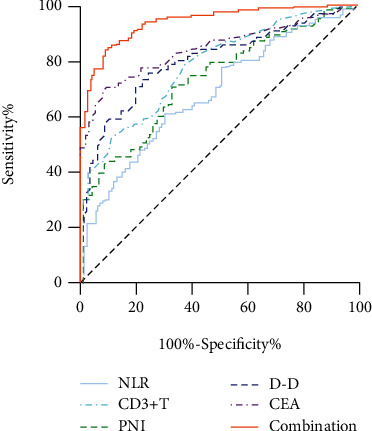
ROC curve of NLR, PNI, D-D, CD3^+^ T, and CEA single and combined detection for colorectal cancer screening.

We further analyzed the ROC curve, and the results showed that the optimal cut-off values of NLR, PNI, D-D, CD3^+^ T, and CEA were 1.95, 51.3, 89 ng/ml, 760 *μ*L^−1^, and 2.73 ng/ml, respectively; The AUC^ROC^ values were 0.684 (95% CI: 0.621-0.747), 0.733 (95% CI: 0.674-0.791), 0.805 (95% CI: 0.754-0.891),0.781 (95% CI:0.728-0.835), and 0.847 (95% CI: 0.804-0.891), respectively. The sensitivity was 60.43%, 71.66%, 75.84%, 53.48%, and 70.59%, respectively. The specificity was 70.00%, 65.00%, 76.00%, 89.00%, and 91.00%, respectively. Youden index is 0.30, 0.37, 0.52, 0.42, and 0.62, respectively. When the five indicators were combined for screening colorectal cancer, AUC^ROC^ reached the maximum value of 0.943 (95% CI: 0.910-0.967), and the sensitivity, specificity, and Youden index were 84.49%, 91.00%, and 0.75, respectively, which were all higher than each single indicator, suggesting that the screening efficacy was superior to that of single detection (*P* < 0.001, Table 2, Figure 1).

### 3.3. The Association of NLR, PNI, D-D, CD3^+^ T, and CEA Levels with the Risk of CRC

To assess the importance of NLR, PNI, D-D, CD3^+^ T, and CEA levels for the screening of CRC, we obtained the crude odds ratio (OR) after logistic regression analysis. To exclude the possible effects of age and sex, we got adjusted odds ratio (ORa) after adjustment for age and sex: CEA (>2.73 ng/ml) was 20.525 (95% CI: 6.817-61.799), PNI (≤51.3) was 3.227 (95% CI: 1.276-8.165), D-D (>89 ng/ml) was 3.499 (95% CI: 1.416-8.645), and CD3^+^ T (≤760 *μ*L^−1^) was 9.514 (95% CI: 3.519-25.725). The results showed that differences in age and sex did not change the screening value of NLR, PNI, D-D, CD3 + T, and CEA for CRC; D-D and CEA were positively correlated with the risk of CRC, while PNI and CD3^+^ T were negatively correlated with the risk of CRC (*P* < 0.05, Table 3).

### 3.4. The Relationship between NLR, PNI, D-D, CD3^+^ T, and CEA Levels with the Clinicopathological Characteristics of CRC Patients

According to the optimal cut-off values determined by the ROC curve, colorectal cancer patients were divided into the high and low groups to evaluate its relationship with clinicopathological characteristics. The high group included CEA > 2.73 ng/ml, NLR > 1.95, PNI > 51.3, D − D > 89 ng/ml, and CD3^+^ T > 760 *μ*L^−1^, while the low group was opposite. The results in Table 4 show that age, differentiation degree, TNM stage, T stage, N stage, nerve invasion ,and distant metastasis were significantly different between the high CEA group and the low CEA group (*P* < 0.05), but there was no significant correlation with other factors, such as tumor size and vascular invasion (*P* > 0.05).Compared with the high CD3^+^ T group in terms of age and tumor location, the difference between the low CD3^+^ T group and the high CD3^+^ T group was statistically significant (*P* < 0.05), and there was no significant correlation with TNM staging, distant metastasis, etc. The high NLR group and low PNI group were only related to tumor size (*P* < 0.05), and the high D-D group was significantly correlated with age, differentiation degree, and tumor size (*P* < 0.05) but not significantly correlated with TNM staging, distant metastasis, etc. (*P* > 0.05).

## 4. Discussion

Colorectal cancer is the most common malignant tumor of the digestive system in the world, with high morbidity and mortality. According to statistics, there were 1.88 million new cases of colorectal cancer and 910,000 deaths worldwide in 2020 [1]. In recent years, with changes in people's diet and lifestyle, as well as the long-term lack of early detection and early treatment of cancer in China, the incidence and fatality rate of colorectal cancer in China have been on the rise [13]. Of course, the insidious onset of colorectal cancer and lack of obvious specific symptoms are also important reasons, which make most patients diagnosed in the middle and advanced stages of the disease, lose the best treatment opportunity. The 5-year survival rate for patients with early colorectal cancer can reach 90.1%, while the 5-year survival rate for metastatic patients drops to 11.7% [14]. Early screening and early diagnosis of colorectal cancer remain a huge challenge for clinicians. Therefore, accurate, noninvasive, and cost-effective biomarkers are urgently needed to aid in the early screening and clinical treatment of colorectal cancer. This study showed that the NLR, D-D, and CEA levels in colorectal cancer patients were significantly higher than those in the control group, while the PNI and CD3^+^ T levels were significantly lower than those in the control group, which was basically consistent with the results of previous studies [15–18], suggesting that these five indicators are closely related to colorectal cancer. We also assessed the relationship between NLR, PNI, D-D, CD3^+^ T, and CEA and CRC risk by adjusting the OR for age and sex and found that D-D and CEA were positively correlated with the risk of CRC, while PNI and CD3^+^ T were negatively correlated with it. Further studies found that the combined detection of NLR, PNI, D-D, CD3^+^ T, and CEA was significantly better than a single indicator for colorectal cancer screening and significantly improved the screening accuracy.

As a nonspecific tumor-associated antigen, the increased expression of CEA is more common in gastrointestinal malignancies. Our results showed that high CEA was significantly correlated with the differentiation degree, TNM stage, T stage, lymph node metastasis, nerve invasion, and distant metastasis of colorectal cancer. This also verifies why CEA is the only tumor marker recommended as routine screening for colorectal cancer in the National Comprehensive Cancer Network guidelines [19]. Our results also showed that CEA had the best screening efficiency for colorectal cancer among all single indicators, with an AUCROC of 0.847 and specificity up to 91.00%, which is similar to the results reported by Zhang et al. [20] and Huang et al. [21]. However, our results showed that CEA sensitivity was only 70.59%. Therefore, we believe that it is necessary to combine other potential clinical blood markers to reduce the rate of missed diagnosis and misdiagnosis in colorectal cancer screening.

As the most direct biomarker of the inflammatory response in the body, NLR has been found to be related to the diagnosis and prognosis of many malignant diseases, including colorectal cancer, gastric cancer, and breast cancer [22–24]. Our study found that NLR has a good screening effect on colorectal cancer, with an AUCROC of 0.684 and an optimal cut-off value of 1.95, which is similar to the report of Li et al. [25]. PNI is mainly used to evaluate the nutritional status of patients with gastrointestinal malignant tumors and is calculated based on serum albumin concentration and total lymphocyte count. There are many reports on the prognostic role of PNI in colorectal cancer [7, 26], but its significance in the screening and diagnosis of colorectal cancer is rarely discussed. Our results show that its AUCROC is 0.733, indicating that PNI has a high diagnostic accuracy for colorectal cancer and is a good potential screening marker. T lymphocyte subsets are important indicators of cellular immune function and play an important role in antitumor immunity. We found that the levels of CD3+ T, CD4+T, and CD4+/CD8+ in the peripheral blood of patients with colorectal cancer were significantly lower than those in the control group, while the level of CD8^+^T was significantly higher, indicating that the cellular immune function of patients with colorectal cancer was inhibited, which was basically consistent with previous reports [27]. This change is in line with the immune escape mechanism of tumors, and colorectal cancer cells may suppress the host immune system through the Fas/FasL pathway to produce immunosuppression [28]. Further analysis of the screening efficiency of CD3 + T lymphocytes showed that its AUC^ROC^ was 0.781, indicating that it was also a relatively reliable blood marker. In addition, there are few studies on the screening of T lymphocyte subsets for colorectal cancer; so, it has high research value. Thrombosis is a common complication of various malignant tumors and has been the second leading cause of death in tumor patients [29].

The pathogenesis of thrombosis is complex, among which hypercoagulability is one of the important reasons. Some studies have found that hypercoagulability plays an important role in tumor angiogenesis, invasion, and metastasis in patients with malignant tumors [30]. D-D is a recognized indicator of coagulation and fibrinolysis activity, which can indirectly judge thrombus activity and is a reliable indicator to evaluate the activation degree of the blood coagulation system. It has been widely accepted that high plasma D-D levels are closely associated with poor prognosis in colorectal cancer patients [16, 31, 32], but the screening value of D-D has been ignored. The results of this study show that the D-D level is a very valuable marker for colorectal cancer screening in single detection. D-D has an AUCROC of 0.805, a sensitivity of 75.84%, and a specificity of 76.00%, which is second only to CEA in diagnostic efficacy. In addition, we found that high plasma D-D level was significantly correlated with age, differentiation degree, and tumor size in colorectal cancer patients, but not with tumor stage and metastasis, which was different from relevant reports [16]. This difference may be related to different sample sizes and different sources of research objects, and it is necessary to further increase the sample data for analysis and verification.

To further improve the accuracy of colorectal cancer screening, this study combined five blood indicators. Surprisingly, our results showed that the combined detection of NLR, PNI, D-D, CD3+ T, and CEA for colorectal cancer screening had an AUCROC of 0.943, a sensitivity of 84.49%, a specificity of 91.00%, and a Youden index of 0.75, and its screening efficacy was significantly superior to that of a single detection. A retrospective study used the combined detection of CEA, CA199, CA125, and CA724 tumor markers in the diagnosis of CRC, but the sensitivity was only 66.67% and the specificity was only 76% [33]. Their screening efficiency was significantly lower than that of the combination of the five indicators in this study. Another study included 664 CRC patients and healthy subjects, respectively, and combined the inflammatory indicators NLR and LMR with CEA, but the AUC^ROC^ of CRC detected by the three combined was only 0.892 [25], which was also lower than our results. Therefore, we believe that NLR, PNI, D-D, CD3^+^ T, and CEA should be detected together to improve the accuracy of colorectal cancer screening.

Of course, this study still has certain limitations. The sample size of this study is small, and due to the single center and retrospective nature of the study, selection bias cannot be completely excluded. More large-sample and multicenter studies are needed to determine its authenticity and reliability.

In conclusion, NLR, PNI, D-D, CD3^+^ T, and CEA are all potential biomarkers for screening colorectal cancer, and the combined application of these five markers in the clinical screening of colorectal cancer has high diagnostic value and can play the role of complementary advantages and mutual correction to improve the overall screening accuracy of colorectal cancer.

## Figures and Tables

**Table 1:** Comparison of NLR, PNI, D-D, CEA, T lymphocyte subsets, and related basic data between the two groups.

Characteristics	Study group (*N* = 187)	Control group (*N* = 100)	*Z*/*X*^2^	*P*
Sex				
Male	116 (67.1)	57 (32.9)	0.406	0.689
Female	71 (62.3)	43 (37.7)		
Age (yr)	63 (51-69)	38 (31-48)	10.526	<0.001
NLR	2.15 (1.66-3.13)	1.69 (1.28-2.15)	5.135	<0.001
PNI	49.4 (45.0-51.9)	52.8 (49.5-55.2)	6.488	<0.001
D-D (ng/ml)	139 (90-278)	58 (42-89)	8.504	<0.001
CEA (ng/ml)	4.90 (2.33-12.80)	1.57 (1.08-2.25)	9.690	<0.001
CD3^+^ T (*μ*L^−1^)	728 (420-1028)	1332 (864-1805)	7.855	<0.001
CD4^+^T (*μ*L^−1^)	612 (454-800)	868 (559-1158)	5.573	<0.001
CD8^+^T (*μ*L^−1^)	732 (448-1180)	538 (408-780)	3.495	<0.001
CD4^+^/CD8^+^	1.34 (1.13-1.67)	1.53 (1.21-1.97)	2.224	0.026

**Table 2:** Comparison of single and combined detection in CRC screening.

Indicators	AUC^ROC^	95% CI	*P*	Cut-off value	Sensitivity (%)	Specificity (%)	Youden index
CD3^+^ T	0.781	0.728-0.835	<0.001	760	53.48	89.00	0.42
NLR	0.684	0.621-0.747	<0.001	1.95	60.43	70.00	0.30
PNI	0.733	0.674-0.791	<0.001	51.3	71.66	65.00	0.37
D-D	0.805	0.754-0.855	<0.001	89	75.84	76.00	0.52
CEA	0.847	0.804-0.891	<0.001	2.73	70.59	91.00	0.62
Combination	0.943	0.910-0.967	<0.001		84.49	91.00	0.75

AUC^ROC^: area under receiver operating characteristic curve; CI: confidence interval.

**Table 3:** The association of NLR, PNI, D-D, CD3^+^ T, and CEA levels with the risk of CRC.

Indicators	OR	95% CI	*P*	ORa^∗^	95% CI	*P*
CEA (>2.73 ng/ml)	23.568	9.358-59.354	<0.001	20.525	6.817-61.799	<0.001
NLR (>1.95)	1.500	0.676-3.327	0.318	1.184	0.481-2.915	0.714
PNI (≤51.3)	2.597	1.182-5.707	0.017	3.227	1.276-8.165	0.013
D-D (>89 ng/ml)	7.369	3.293-16.492	<0.001	3.499	1.416-8.645	0.007
CD3^+^ T (≤760 *μ*L^−1^)	11.146	4.379-28.368	<0.001	9.514	3.519-25.725	<0.001

OR: crude odds ratio; ORa: adjusted odds ratio; ^∗^Adjustment for age and sex; CI: confidence interval.

**Table 4:** The relationship between NLR, PNI, D-D, CD3+ T, and CEA levels with clinicopathological characteristics of CRC patients.

Characteristics	CEA (ng/ml)			NLR			PNI			D-D (ng/ml)			CD3^+^ T (*μ*L^−1^)		
	≤2.73	>2.73	*P*	≤1.95	>1.95	*P*	≤51.3	>51.3	*P*	≤89	>89	*P*	≤760	>760	*P*
Sex															
Male	30	86	0.173	41	75	0.131	86	30	0.336	29	87	0.702	66	50	0.311
Female	25	46		33	38		48	23		16	55		35	36	
Age (yr)															
<63	37	55	0.001	42	50	0.094	63	29	0.342	33	59	<0.001	57	35	0.032
≥ 63	18	77		32	63		71	24		12	83		44	51	
Tumor location															
Colon	33	64	0.151	37	60	0.678	66	31	0.255	25	72	0.570	84	13	<0.001
Rectum	22	68		37	53		68	22		20	70		17	73	
Histological type															
Nonspecific adenocarcinoma	54	128	1.000	73	109	0.650	129	53	0.324	44	138	1.000	99	88	0.663
Others	1	4		1	4		5	0		1	4		2	3	
Differentiation degree															
Well+moderate	46	92	0.048	59	79	0.135	97	41	0.486	39	99	0.024	74	64	0.858
Poor	9	40		15	34		37	12		6	43		27	22	
Vascular invasion															
Yes	9	35	0.136	18	26	0.836	31	13	0.84	8	36	0.297	27	17	0.263
No	46	97		56	87		103	40		37	106		74	69	
Nerve invasion															
Yes	12	54	0.013	29	37	0.367	49	17	0.562	16	50	0.966	31	35	0.154
No	43	78		45	76		85	36		29	92		70	51	
Tumor size (cm)															
< 5	44	88	0.068	59	73	0.026	89	43	0.047	38	94	0.019	70	62	0.677
≥ 5	11	44		15	40		45	10		7	48		31	24	
TNM stage															
0, I, II	40	60	0.001	35	65	0.170	72	28	0.911	25	75	0.748	55	45	0.771
III, IV	15	72		39	48		62	25		20	67		46	41	
T stage															
Tis + T1 + T2	21	22	0.001	18	25	0.727	31	12	0.942	15	28	0.059	19	24	0.141
T3 + T4	34	110		56	88		103	41		30	114		82	62	
Lymph node metastasis															
N0	40	61	0.001	36	65	0.234	73	28	0.839	25	76	0.811	56	45	0.670
N1 + N2	15	71		38	48		61	25		20	66		45	41	
Distant metastasis															
Yes	2	20	0.026	10	12	0.548	17	5	0.534	3	19	0.223	12	10	0.957
No	53	112		64	101		117	48		42	123		89	76	

## Data Availability

All the data supporting the findings of the study are available in “Results” point. The databases generated during the current study are not publicly available on the internet but are available from the corresponding author on request.
